# Se Alleviated Pb-Caused Neurotoxicity in Chickens: *SPS2*-*GPx1*-GSH-*IL-2*/*IL-17*-NO Pathway, Selenoprotein Suppression, Oxidative Stress, and Inflammatory Injury

**DOI:** 10.3390/antiox13030370

**Published:** 2024-03-18

**Authors:** Yansheng Li, Jiatian Liang, Chunyu Jiang, Jiawen Cui, Lan Hong, Zhiyu Hao, You Tang, Yuhao Liu, Xun Cui, Xiaohua Teng

**Affiliations:** 1 College of Medicine, Yanbian University, Yanji 133002, China; 2 College of Animal Science and Technology, Northeast Agricultural University, Harbin 150030, China; 3 Electrical and Information Engineering College, Jilin Agricultural Science and Technology University, Jilin 132101, China

**Keywords:** lead, selenium, selenoproteins, oxidative stress, inflammation, *SPS2*–*GPx1*–GSH–*IL-2*/*IL-17*–NO pathway

## Abstract

Lead (Pb), a heavy metal environmental pollutant, poses a threat to the health of humans and birds. Inflammation is one of the most common pathological phenomena in the case of illness and poisoning. However, the underlying mechanisms of inflammation remain unclear. The cerebellum and the thalamus are important parts of the nervous system. To date, there have been no reports of Pb inducing inflammation in animal cerebellums or thalami. Selenium (Se) can relieve Pb poisoning. Therefore, we aimed to explore the mechanism by which Se alleviates Pb toxicity to the cerebellums and thalami of chickens by establishing a chicken Pb or/and Se treatment model. Our results demonstrated that exposure to Pb caused inflammatory damage in cerebellums and thalami, evidenced by the characteristics of inflammation, the decrease in anti-inflammatory factors (*interleukin (IL)-2* and *interferon-γ (INF-γ)*), and the increase in pro-inflammatory factors (*IL-4*, *IL-6*, *IL-12β*, *IL-17*, and nitric oxide (NO)). Moreover, we found that the *IL-2*/*IL-17*–NO pathway took part in Pb-caused inflammatory injury. The above findings were reversed by the supplementation of dietary Se, meaning that Se relieved inflammatory damage caused by Pb via the *IL-2*/*IL-17*–NO pathway. In addition, an up-regulated oxidative index malondialdehyde (MDA) and two down-regulated antioxidant indices (glutathione (GSH) and total antioxidant capacity (TAC)) were recorded after the chickens received Pb stimulation, indicating that excess Pb caused an oxidant/antioxidant imbalance and oxidative stress, and the oxidative stress mediated inflammatory damage via the GSH–*IL-2* axis. Interestingly, exposure to Pb inhibited four *glutathione peroxidase (GPx)* family members (*GPx1*, *GPx2*, *GPx3*, and *GPx4*), three *deiodinase (Dio)* family members (*Dio1*, *Dio2*, and *Dio3*), and fifteen other selenoproteins (*selenophosphate synthetase 2 (SPS2)*, *selenoprotein (Sel)H*, *SelI*, *SelK*, *SelM*, *SelO*, *SelP1*, *SelPb*, *SelS*, *SelT*, *SelU*, and selenoprotein (Sep)n1, *Sepw1*, *Sepx1*, and *Sep15*), suggesting that Pb reduced antioxidant capacity and resulted in oxidative stress involving the *SPS2*–*GPx1*–GSH pathway. Se supplementation, as expected, reversed the changes mentioned above, indicating that Se supplementation improved antioxidant capacity and mitigated oxidative stress in chickens. For the first time, we discovered that the *SPS2*–*GPx1*–GSH–*IL-2*/*IL-17*–NO pathway is involved in the complex inflammatory damage mechanism caused by Pb in chickens. In conclusion, this study demonstrated that Se relieved Pb-induced oxidative stress and inflammatory damage via the *SPS2*–*GPx1*–GSH–*IL-2*/*IL-17*–NO pathway in the chicken nervous system. This study offers novel insights into environmental pollutant-caused animal poisoning and provides a novel theoretical basis for the detoxification effect of Se against oxidative stress and inflammation caused by toxic pollutants.

## 1. Introduction

Lead (Pb) is a toxic and non-biodegradable heavy metal. Its widespread uses in fields, such as the chemical industry, shipbuilding, cables, storage batteries, gasoline, and radiation protection, lead to the release of Pb into the environment, causing extensive environmental pollution, human exposure, and significant public health problems in numerous regions across the globe [1]. In Vilnius, Lithuania, shooting range workers had Pb concentrations up to 45.8 μg/dL in their blood and showed symptoms of fatigue, dizziness, memory impairment, nausea, sleep disturbance, and balance disorder [2]. In Patna, India, a high blood Pb level (>5 μg/dL) was found in 37 children (0–12 years old) living near a Pb battery recycling workshop, and most of the children suffered hyperactivity, lethargy, and headaches [3]. Pb pollution also threatens birds. Pb was found to be accumulated in the food of bar-headed geese (*Anser indicus*), black-necked cranes (*Grus nigricollis*), and red-skinned ducks (*Tadorna ferruginea*) in the Caohai Plateau Wetland, Guizhou, China, and the birds were considered to be suffering a high risk of Pb exposure [4]. In the Pb-polluted area of Baiyin, China, Pb accumulation was found in tree sparrow feathers and bones, and the growth of tree sparrows was slowed down [5]. The cerebellum (Ce) and the thalamus (Th) are both integral components of the nervous system. The Ce can coordinate body movement and maintain body balance. The Th is the center of somatic and visceral sensation. It was reported that Pb exposure damaged rat cerebellums (Ces) [6] and thalami (Thi) [7]. Thus, we wanted to explore the mechanism of Pb-induced neurotoxicity in chickens by studying their Ces and Thi.

Two studies found that heavy metals can cause inflammatory damage in the Ces and Thi of chickens. Sun et al. (2017) mentioned that arsenic exposure induced cerebellar and thalamic inflammatory pathological changes and inflammatory responses [8]. Excess copper and arsenic treatment led to thalamic inflammatory injury [9]. Nevertheless, whether Pb can result in inflammation in Ces and Thi is still unknown. Interestingly, recent studies found that *interleukin (IL)-2*, *IL-4*, *IL-6*, *IL-12β*, *IL-17*, *interferon-γ* (*INF-γ*), and nitric oxide (NO) participate in heavy metal-caused inflammatory responses and inflammatory damage. Pb treatment led to inflammatory damage with abnormal *IL-2*, *IL-4*, and *IL-12β* expression in chicken testes [10]. Under cadmium (Cd) exposure, mRNA levels of *IL-4*, *IL-6*, and *INF-γ* were changed, and inflammatory injury occurred in pig small intestines [11]. In a rat model of ear inflammation induced by hapten dinitrochlorobenzene, after oral Cd, inflammatory damage was aggravated with higher *IL-17* in ears [12]. Wu et al. (2021) found that Pb challenge induced an inflammatory response via the detection of *IL-6* and NO in RAW264.7 macrophages [13]. Therefore, we hypothesized that Pb stress can affect the above seven factors and lead to inflammatory damage in both chicken Ces and Thi. Se is an essential micronutrient for organisms. Researchers reported that Se can resist environmental pollutant-caused inflammation. For example, a reduction in *IL-2*, an elevation in *IL-4* and *IL-12β*, and inflammatory damage caused by Pb were mitigated by Se in chicken testes [10] and hearts [14]. Se treatment alleviated bisphenol A-caused *IL-6* and *IL-17* elevation and inflammatory response in IPEC-J2 cells [15], as well as relieved Pb-caused *IL-6* and NO increase and inflammatory response in RAW264.7 macrophages [13]. Thus, we assumed that Se has an anti-inflammatory function under Pb stress in chicken Ces and Thi through the measurement of *IL-2*, *IL-4*, *IL-6*, *IL-12β*, *IL-17*, *INF-γ*, and NO. 

Heavy metal toxicity also can cause oxidative stress in Ces and Thi. Oxidative stress and tissue lesions in Thi occurred in chickens receiving excess copper and arsenic, and glutathione (GSH) and total antioxidant capacity (TAC) decreased, while malondialdehyde (MDA) increased [16]. Cd poisoning led to oxidative stress with elevated MDA and tissue damage in chicken Ces [17]. Hence, we inferred that Pb can down-regulate GSH and TAC, up-regulate MDA, and lead to oxidative stress in chicken Ces and Thi. Additionally, Salaramoli et al. (2023) demonstrated that Se ameliorated oxidative stress in the brains of rats with Parkinson’s disease by up-regulating TAC and down-regulating MDA [18]. Se supplement relieved Cd-induced cerebral oxidative stress via increasing GSH and TAC and decreasing MDA in rabbits [19]. However, whether Se has an ameliorative effect on Pb-induced GSH and TAC reductions and Pb-induced MDA elevation is still unclear in animal Ces and Thi. 

Selenoproteins (such as glutathione peroxidase (GPx) family (including GPx1, GPx2, GPx3, and GPx4), the deiodinase (Dio) family (including Dio1, Dio2, and Dio3), selenophosphate synthetase 2 (SPS2), selenoprotein (Sel)H, SelI, SelK, SelM, SelO, SelP1, SelPb, SelS, SelT, SelU, and selenoprotein (Sep)n1, Sepw1, Sepx1, and Sep15) are Se-contained proteins that are closely associated with the exercise of Se biological functions [20]. Under Pb treatment conditions, two GPx family members (GPx1 and GPx4), SelK, and Sep15 were down-regulated in mouse livers [21], as well as four GPx family members (GPx1-4), three Dio family members (Dio1-3), SPS2, SelH, SelI, SelK, SelM, SelO, SelS, SelT, SelU, SelP1, SelPb, Sepn1, Sepx1, and Sep15, declined in chicken brainstems [22]. A recent study confirmed that Cd resulted in abnormal selenoprotein mRNA levels (three GPx family members (GPx1-3), SelH, SelI, SelK, SelM, SelO, SelS, SelT, SelP1, Sepx1, and Sep15 were decreased) in chicken Ces [17]. However, to the best of our knowledge, there is no research on the effect of Pb on the above 22 selenoproteins in Ces and Thi. Accordingly, we postulated that the decline in selenoproteins was involved in the molecular mechanism of Pb poisoning. Additionally, Chen et al. (2024a) reported that Se reversed twenty-two Pb-decreased selenoproteins (the GPx family (GPx1-4), the Dio family (Dio1-3), SPS2, SelH, SelI, SelK, SelM, SelO, SelS, SelT, SelU, SelP1, SelPb, Sepn1, Sepw1, Sepx1, and Sep15) in chicken brainstems [22]. However, whether the above 22 selenoproteins participated in the molecular mechanism by which Se antagonized Pb toxicity is unknown. 

Thereby, our study aimed to establish a chicken Pb or/and Se model in order to explore the complex mechanism of Se-counteracted Pb toxicity in chicken Ces and Thi from the perspective of inflammation, oxidative stress, and selenoproteins, using microstructural observation, kit assays, qRT-PCR technology, and integrated biomarker response (IBR) value calculation. The current study was expected to advance the knowledge of Pb poisoning and furnish evidence for the detoxification of Se.

## 2. Materials and Methods

### 2.1. Animal Model

In this experiment, all the chickens (purchased from Weiwei company in Harbin, China) were raised in the Experimental Animal Center of the College of Veterinary Medicine, Northeast Agricultural University (Harbin, China), and all the procedures were approved by the Northeast Agricultural University Institutional Animal Care and Use Committee (approval number: NEAUEC20210211). After a seven-day adaptation period, one hundred and eighty healthy 1-day-old male Hyline chickens were randomly assigned to 4 groups with 45 chickens in each group. Three replicates were set up in each group with 15 chickens in each replicate. The diet provided for each group was as follows. The control and Pb groups received a standard commercial diet (containing 0.49 mg/kg Se). The composition of the chicken standard commercial diet is shown in Table S1. The Se and Se/Pb groups received a Na_2_SeO_3_ (Tianjinzhiyuan Chemical Reagent Co., Ltd., Tianjin, China)-supplemented standard diet (containing 1.00 mg/kg Se). Drinking water provided for each group was as follows. The control and Se groups were provided with Pb-free drinking water. The Pb and Se/Pb groups were provided with (CH_3_COO)_2_Pb (Tianjinzhiyuan Chemical Reagent Co., Ltd., Tianjin, China)-supplemented drinking water (containing 350 mg/L Pb). The selection of 350 mg/L Pb was based on the following. Meng (2003) recommended the use of 1/5 of the median lethal dose (LD_50_) as an experimental dose of poison in animal poisoning experiments [23]. Vengris and Mare (1974) reported an LD_50_ of 320 mg/kg body weight for Pb in chickens [24]. Thus, 1/5 of LD_50_ for Pb in chickens was 64.39 mg/kg body weight, and we chose 350 mg/L Pb in drinking water (equivalent to 64.39 mg/kg Pb/body weight). In addition, other studies on Pb poisoning in chickens also supported our choice of Pb dose. We found that 350 mg/L Pb in drinking water was also used in chicken Pb poisoning studies conducted by Wang et al. (2021a) [10] and Li et al. (2017) [25]. Additionally, in another study conducted by Cai et al. (2021) [26], we found that 160 mg/kg body weight lead acetate (gavage, equivalent to 101.92 mg/kg Pb/body weight, 3/10 of the LD_50_) was used in a chicken Pb poisoning study. In our experiment, the chickens were housed and kept under the following environmental conditions: The chickens were provided with ad libitum access to feed and water. The temperature was 32–34 °C for 1–2 days of age and 30–32 °C for 3–7 days of age and was dropped 2 °C every week from the second week until it was constant at 19–22 °C in the sixth week. Humidity was 60–65% for 1–7 days of age and 55–60% for after 7 days of age. Light conditions were 22 h of light for 1–3 days of age, 21 h of light for 4–7 days of age, and 12 h of light or dark cycle after 7 days of age.

### 2.2. Sample Collection

On days 30, 60, and 90 of our experiment, 15 chickens in each group (5 birds per replicate) were randomly selected and were euthanized with pentobarbital. The cerebellar and thalamic tissues were rapidly removed and rinsed with cold sterile deionized water. The obtained cerebellar and thalamic tissues were treated as follows. Some of the samples were cut into 1 cm^3^ pieces and were fixed with 4% (*v*/*v*) formaldehyde solution for microstructural observation. Some of the samples were homogenized with normal saline for the detection of 3 oxidative stress-related indexes (GSH, TAC, and MDA) and an inflammation-related factor NO. Some of the samples were immediately put into liquid nitrogen and were stored at –80 °C for subsequent analysis of mRNA expressions of 22 selenoproteins (*GPx1-4*, *Dio1*-*3*, *SPS2*, *SelH*, *SelI*, *SelK*, *SelM*, *SelO*, *SelS*, *SelT*, *SelU*, *SelPb*, *Sepn1*, *SelP1*, Selw1, *Sepx1*, and *Sep15*) and 6 inflammation-related factors (*IL-2*, *IL-4*, *IL-6*, *IL-12β*, *IL-17*, and *INF-γ*).

### 2.3. The Observation of Microstructure 

The fixed chicken cerebellar and thalamic samples were dehydrated with a gradient of alcohol (concentrations were 70%, 80%, 90%, 95%, and 100%, respectively). Each dehydration step lasted for 2 h. The dehydrated tissues were embedded in paraffin. The obtained wax blocks were cut into 5–10 μm slices using a slicer (Jinhua Wireless Power Plant, Jinhua, China). After that, the slices were stained with hematoxylin and eosin, sealed with neutral resin, and scanned using a digital slice scanning system (WINMEDIC, Jinan, China). The above steps were performed by Servicebio Technology Co., Ltd. (Wuhan, China). Finally, chicken cerebellar and thalamic microstructures were observed using ZYFViewer software (version 1.0; Shandong Zhiying Medical WINMEDIC Technology Co., Ltd., Jinan, China).

### 2.4. The Determination of GSH, TAC, MDA, and NO

The obtained tissue homogenates of chicken Ces and Thi were used to detect GSH, MDA, and NO contents, as well as TAC activity. A GSH assay kit (Spectrophotometric method, number: A006-1-1), TAC assay kit (number: A014-2-2), MDA assay kit (TBA method, number: A003-1-2), and NO assay kit (Microwell plate method, number: A013-2-1) were used for these measurements. All the kits were purchased from Nanjing Jiancheng Bioengineering Institute (Nanjing, China). All the experimental operations were carried out in accordance with the manufacturer’s instructions.

### 2.5. The Detection of Selenoproteins and Inflammation-Related Genes

#### 2.5.1. Primer Acquisition and Total RNA Extraction

Primer sequence information on 22 selenoproteins and 6 inflammation-related factors, as well as *β-actin*, were obtained from NCBI (https://www.ncbi.nlm.nih.gov/, accessed on 12 January 2023), and the primers were synthesized by Sangon Biotechnology (Shanghai, China). *β-actin* was chosen as the reference gene in our experiment. Specific information on all the above primers is listed in Table 1. 

Total RNA was extracted from chicken Ce and Th samples (stored at −80 °C) with TRIzol reagent (Takara, Kyoto, Japan). The brief steps were as follows. The samples were taken out from a −80 °C refrigerator and were put into tubes. Then, 1 mL of TRIzol and 0.4 mL of chloroform were added into each tube, and the tubes were shaken vigorously and placed at room temperature for 9 min. The tubes were centrifuged (12,000× *g*) at 4 °C for 15 min, and the supernatants were transferred into new tubes. An equal volume of isopropyl alcohol as the supernatant was added into the new tubes, followed by centrifugation (12,000× *g*) at 4 °C for 15 min. The supernatants were discarded, and the sediments at the bottom of the new tubes were washed with 1 mL of 75% iced ethanol (the new tubes were centrifuged (10,000× *g*) again at 4 °C for 10 min, and the supernatants were discarded again). Total RNA was obtained for complementary DNA (cDNA).

#### 2.5.2. cDNA Synthesis and PCR Amplification

In our experiment, the OD260/OD280 ratio of obtained total RNA was more than 1.9 and less than 2.1, which met the RNA purity requirement. cDNA synthesis was performed with a FastKing cDNA first-strand synthesis kit, which was purchased from Tiangen Biochemical Technology Co., Ltd. (Beijing, China). The obtained cDNA was then used for PCR amplification. 

The total volume of the PCR amplification system was 20 μL, including cDNA (2 μL), the upstream primers of tested genes (0.6 μL), the downstream primers of tested genes (0.6 μL), SYBR^®^ Premix Ex Taq^TM^ (10 μL, Beijing Think-Far Technology Co., Ltd., Beijing, China), and H_2_O (6.8 μL). PCR amplification was performed using a Real-time PCR instrument (Applied Biosystems, Foster City, CA, USA). Reaction conditions were set at 95 °C for 30 s, at 95 °C for 5 s, and at 60 °C for 34 s. The 2^−ΔΔCt^ method [27,28,29] was used to calculate relative mRNA levels.

### 2.6. Statistical Analysis and IBR Value Calculation

SPSS (version 25.0; Chicago, IL, USA) was used for data analysis. The raw data passed normality and variance homogeneity tests. Two-way ANOVA was performed to analyze whether there were significant differences between different groups at the same time point and between different time points in the same group. *p* < 0.05 was considered statistically significant. The obtained data were visualized as bar charts using prism (version 6.0; Graph-Pad Software, Inc., La Jolla, CA, USA). Each bar chart was presented as mean ± standard deviation (SD) (*n* = 5). Bar charts with different lowercase letters between different groups at the same time point and different capital letters between different time points in the same group were considered significantly different (*p* < 0.05). 

The means of all detected factors (including 22 selenoproteins, 3 oxidative stress indexes, and 7 inflammatory-related factors) were used to further calculate IBR values in order to further assess Pb-caused toxicity on the Ces and Thi of chickens. IBR value calculation formula was as follows: Y_i_ = log (X_i_/X_0_); Z_i_ = (Y_i_ − μ)/σ; A_i_ = Z_i_ − Z_0_; IBR = Σ|A_i_| (X_i_ means the average value of different factors in each group).

## 3. Results

### 3.1. The Symptoms of Chickens 

During the experiment, chickens in the control and Se groups were lively and active, with normal standing and walking conditions. Some chickens in the Pb group showed half-closed eyes, constricted necks, reduced activity, lethargy, disordered movements, signs of blood in stools, and even lying down and not getting up. In the Se/Pb group, some chickens displayed the behaviors of half-closed eyes, contracted necks, reduced activity, and lethargy. Compared with the Pb group, these clinical toxicological symptoms were mitigated.

### 3.2. The Changes in Microstructures in Chicken Ces and Thi 

**Figure 1:**
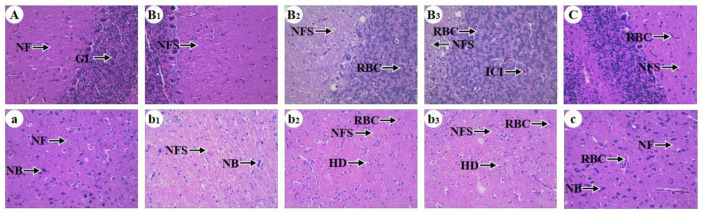
The microstructures of the Ces and Thi of chickens. Magnification: ×400. Uppercase letters represent Ces and lowercase letters represent Thi. (**A**) and (**a**): the 90-day control group; (**B1**) and (**b1**): the 30-day Pb exposure group; (**B2**) and (**b2**): the 60-day Pb exposure group; (**B3**) and (**b3**): the 90-day Pb exposure group; (**C**) and (**c**): the 90-day Se/Pb group. NF: nerve fibers, GL: granular layer, NFS: nerve fiber space, RBC: red blood cell (RBC), ICI: inflammatory cell infiltration, NB: Nissl body, HD: hydropic degeneration.

Chicken Ce and Th microstructures were observed to explore whether Pb can cause inflammatory injury and whether Se can antagonize Pb-caused inflammatory injury. Regarding the Ces, the 90-day control group (Figure 1A) exhibited normal microstructures, characterized by dense nerve fibers (NFs) and a granular layer (GL). The 30-day Pb exposure group (Figure 1B_1_) displayed larger nerve fiber space (NFS) compared with the control group. The 60-day Pb exposure group (Figure 1B_2_) developed larger nerve fiber spaces compared with the 30-day Pb exposure group and red blood cells (RBCs). The 90-day Pb exposure group (Figure 1B_3_) showed larger nerve fiber space and more red blood cells compared with the 60-day Pb exposure group, as well as inflammatory cell infiltration (ICI). The 90-day Se/Pb group (Figure 1C) showed red blood cells and smaller nerve fiber space compared with the 90-day Pb exposure group; on the contrary, it showed larger nerve fiber space compared with the 90-day control group. 

Regarding the Thi, the 90-day control group (Figure 1a) exhibited normal microstructures, characterized by clear Nissl bodies (NBs) and dense nerve fibers. The 30-day Pb exposure group (Figure 1b_1_) showed fewer Nissl bodies and larger nerve fiber spaces compared with the 90-day control group. The 60-day (Figure 1b_2_) and 90-day (Figure 1b_3_) Pb exposure groups both displayed disappeared Nissl bodies and the occurrence of red blood cells, large nerve fiber spaces, and hydropic degeneration (HD). The degree of hydropic degeneration of nerve cells in the 90-day Pb exposure group was more serious compared with the 60-day Pb exposure group. The 90-day Se/Pb group (Figure 1c) showed fewer Nissl bodies compared with the 90-day control group, as well as more dense nerve fibers and fewer red blood cells compared with the 90-day Pb exposure group. 

### 3.3. The Changes in Oxidative Stress-Related Indexes in Chicken Ces and Thi

**Figure 2:**
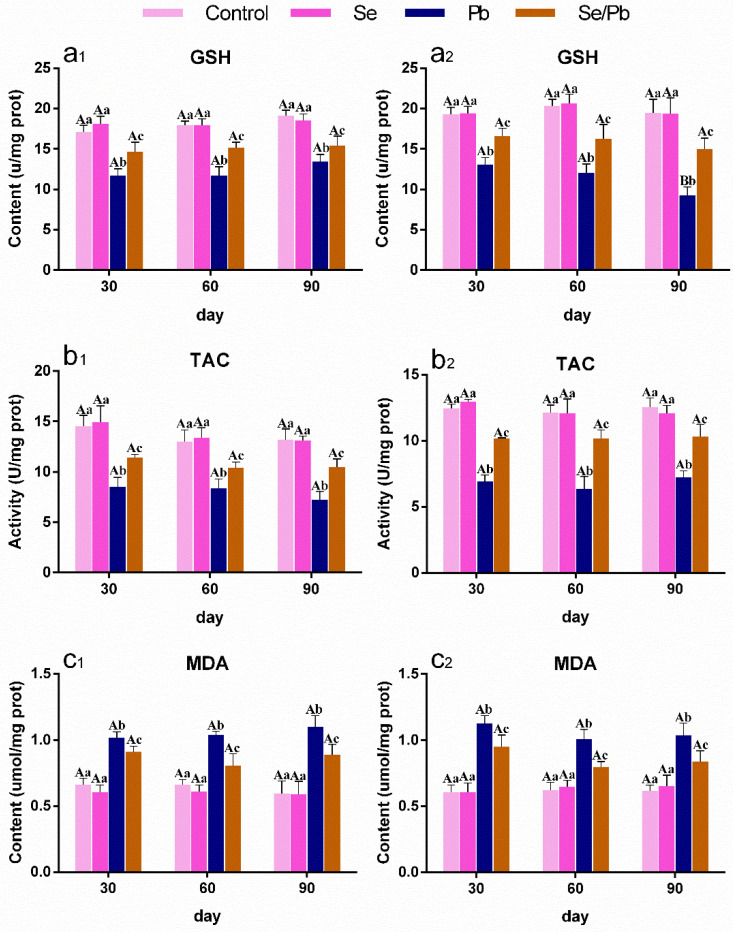
The changes in oxidative stress indexes in chicken Ces and Thi. Number _1_ represents Ces and number _2_ represents Thi. (**a**): GSH; (**b**): TAC; and (**c**): MDA. Different lowercase letters indicate that there are significant (*p* < 0.05) differences among different groups at the same time point, and different capital letters indicate that there are significant (*p* < 0.05) differences among different time points in the same group. Each value is shown as mean ± standard deviation (*n* = 5).

We measured GSH (Figure 2a_1_,a_2_) and MDA (Figure 2c_1_,c_2_) contents, as well as TAC activity (Figure 2b_1_,b_2_) with kits in chicken Ces (presented as number _1_) and Thi (presented as number _2_). As shown in Figure 2, there were no significant (*p* > 0.05) differences between the control group and the Se group in all three factors at the three time points. GSH content and TAC activity decreased significantly (*p* < 0.05) in the Pb group compared with the control and Se groups, on the contrary, MDA content increased significantly (*p* < 0.05) in the Pb group compared with the control and Se groups at all the three time points in chicken Ces and Thi. GSH content and TAC activity in the Se/Pb group were significantly (*p* < 0.05) higher than those in the Pb group and were significantly (*p* < 0.05) lower than those in the control and Se groups. MDA content in the Se/Pb group was significantly (*p* < 0.05) lower than that in the Pb group and was significantly (*p* < 0.05) higher than that in the control and Se groups. In addition, in the Pb group, GSH content in chicken Thi on day 90 was significantly (*p* < 0.05) down-regulated compared with that on days 30 and 60. 

### 3.4. The Changes in Inflammatory-Related Factors in Chicken Ces and Thi

**Figure 3:**
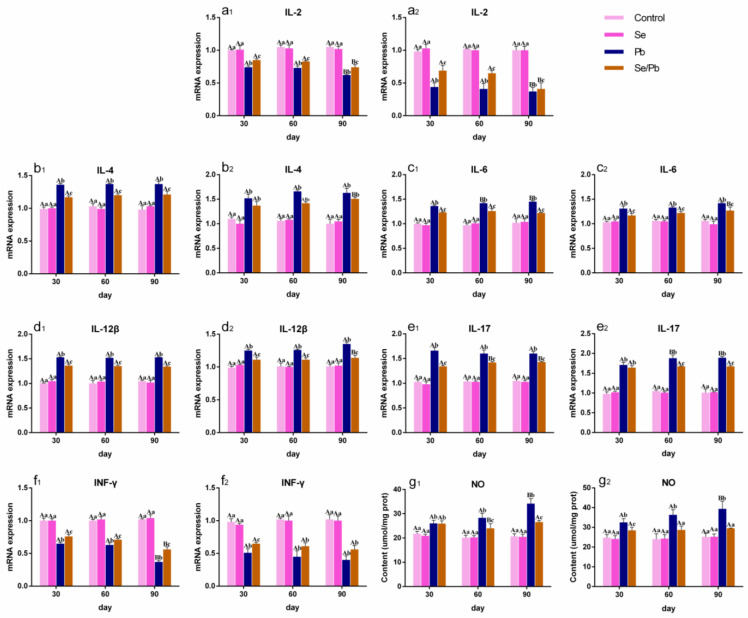
The changes in inflammatory-related factors in chicken Ces and Thi. Number **_1_** represents Ces and number **_2_** represents Thi. (**a**): *IL-2*; (**b**): *IL-4*; (**c**): *IL-6*; (**d**): *IL-12β*; (**e**): *IL-17*; (**f**): *INF-*γ; and (**g**): NO. Different lowercase letters indicate that there are significant (*p* < 0.05) differences between groups at the same time point, and different capital letters indicate that there are significant (*p* < 0.05) differences between different time points in the same group. Each value is shown as mean ± standard deviation (*n* = 5).

In order to explore the inflammatory mechanism of the Pb or/and Se effect on chicken Ces and Thi, mRNA levels of six inflammatory-related genes (including *IL-2* (Figure 3a_1_,a_2_), *IL-4* (Figure 3b_1_,b_2_), *IL-6* (Figure 3c_1_,c_2_), *IL-12β* (Figure 3d_1_,d_2_), *IL-17* (Figure 3e_1_,e_2_), and *INF-γ* (Figure 3f_1_,f_2_)) and the content of NO (Figure 3g_1_,g_2_) were detected under three time points using qRT-PCR and with kits, respectively, and the results are shown in Figure 3. No significant (*p* > 0.05) differences in any of the factors were found between the control group and the Se group at all three time points. *IL-2* and *INF-γ* were significantly (*p* < 0.05) down-regulated; on the contrary, *IL-4*, *IL-6*, *IL-12β*, *IL-17*, and NO were significantly (*p* < 0.05) up-regulated at three time points in the Pb group compared with those in the control and Se groups in both Ces and Thi. *IL-2* and *INF-γ* were significantly (*p* < 0.05) up-regulated; on the contrary, *IL-4*, *IL-6*, *IL-12β*, *IL-17*, and NO were significantly (*p* < 0.05) down-regulated at three time points in the Se/Pb group compared with those in the Pb group in both Ces and Thi, except for the insignificant (*p* > 0.05) up-regulation of *IL-2* on day 90 and *INF-γ* on days 60 and 90 in Thi, as well as the insignificant (*p* > 0.05) down-regulation of *IL-4* on days 30 and 90, *IL-17* on day 30 in Thi, and NO on day 30 in Ces. *IL-2* and *INF-γ* were significantly (*p* < 0.05) lower; on the contrary, *IL-4*, *IL-6*, *IL-12β*, *IL-17*, and NO were significantly (*p* < 0.05) higher at three time points in the Se/Pb group compared with those in the control and Se groups in both Ces and Thi. Additionally, regarding the Pb group, *IL-2* and *INF-*γ mRNA levels on day 90 were significantly (*p* < 0.05) lower than those on days 30 and 60; on the contrary, NO content on day 90 was significantly (*p* < 0.05) higher than that on days 30 and 60 in both Ces and Thi. *IL-6* mRNA levels in Ces and *IL-17* mRNA levels in Thi on days 60 and 90 were significantly (*p* < 0.05) higher than those on day 30. 

### 3.5. The Changes in Selenoprotein in Chicken Ces and Thi

In order to explore whether selenoproteins took part in the molecular mechanism of Se mitigating Pb poisoning, we examined transcriptional levels of 22 selenoproteins at three time points (Table 2). There were no significant (*p* > 0.05) differences between the control group and the Se group in chicken Ces and Thi, except for a significant (*p* < 0.05) up-regulation of *SelPb* on day 30 in the Se group compared with the control group in Ces. All the detected 22 selenoproteins in the Pb group were significantly (*p* < 0.05) lower than those in the control and Se groups at the three time points. In the Se/Pb group, 22 selenoproteins were significantly (*p* < 0.05) higher than those in the Pb group and were significantly (*p* < 0.05) lower than those in the control group at all three time points, except that on day 30, *GPx3* was lower (*p* > 0.05) than that in the control group and *GPx4* was higher (*p* > 0.05) than that in the Pb group in Ces. In addition, regarding the Pb group, *GPx2*, *GPx4*, *Sepw1*, and *Sep15* in Ces, as well as *SelM*, *SelS*, *SelP1*, and *Sepw1* in Thi, on days 60 and 90 were down-regulated significantly (*p* < 0.05) compared with those on day 30. *SelI* and *Sepx1* in Ces and *Sepn1* in Thi mRNA levels on day 90 were down-regulated significantly (*p* < 0.05) compared with those on days 30 and 60. *GPx1* in Ces and *Dio2*, *Dio3*, and *SelT* in Thi on day 90 were down-regulated significantly (*p* < 0.05) compared with those on day 30. With the extension of Pb treatment time, *SPS2* in Ces and *SelH*, *SelO*, *SelU*, and *SelPb* in Thi were down-regulated significantly (*p* < 0.05).

### 3.6. The Changes in IBR Values of Selenoproteins, Oxidative Stress Indexes, and Inflammatory-Related Factors in Chicken Ces and Thi

**Figure 4:**
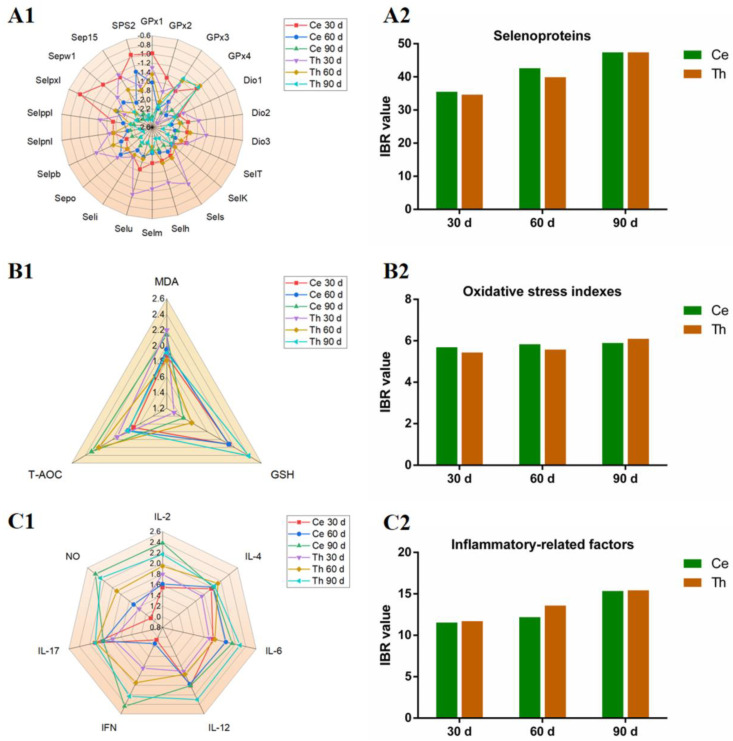
The response levels and the IBR values of selenoproteins, oxidative stress indexes, and inflammatory-related factors. Ce: cerebellum; Th: thalamus. (**A1**): The response levels of selenoproteins; (**A2**): the IBR values of selenoproteins; (**B1**): the response levels of oxidative stress indexes; (**B2**): the IBR values of oxidative stress indexes; (**C1**): the response levels of inflammatory-related factors; and (**C2**): the IBR values of inflammatory-related factors.

IBR value can be used to evaluate the toxic effects of environmental pollutants on organisms. We calculated the response levels of all the measured factors, as well as IBR values of selenoproteins, oxidative stress indexes, and inflammatory-related factors, in order to compare the differences in Pb toxicity in the Ces and Thi of chickens. As shown in Figure 4, we found that the response levels of *GPx1*, *GPx2*, *GPx4*, *Sepx1*, *Sepw1*, *SPS2*, *IL-2*, *INF-γ*, NO, MDA, and TAC on day 30; the response levels of *Dio1*, *SelM*, *SelO*, *SelP1*, *Sepw1*, *SPS2*, *IL-2*, *IL-4*, *INF-γ*, *IL-17*, NO, and TAC on day 60; and the response levels of *GPx1*, *GPx2*, *Dio1*, *Dio2*, *Dio3*, *SelH*, *SelI*, *SelK*, *SelO*, *SelS*, *SelT*, *SelU*, *SelPb*, *Sepn1*, *Sep15*, *IL-4*, *IL-6*, *IL-12β*, *IL-17*, and GSH on day 90 in Thi were higher than those in Ces on days 30, 60, and 90, respectively. The IBR values of selenoproteins (Figure 4A2), oxidative stress indexes (Figure 4B2), and inflammatory-related factors (Figure 4C2) increased with time both in chicken Ces and Thi. The IBR values of selenoproteins on days 30, 60, and 90, the IBR values of oxidative stress indexes on days 30 and 60, and the IBR value of inflammatory-related factors on day 60 in Ces were higher than those in Thi; on the contrary, the IBR value of oxidative stress indexes on day 90 and the IBR values of inflammatory-related factors on days 30 and 90 in Ces were lower than those in Thi.

## 4. Discussion

Pb is toxic to animals. In our experiment, Pb-exposed chickens exhibited neurological symptoms, such as half-closed eyes, constricted necks, reduced activity, lethargy, and disordered movements. Our findings demonstrated that Pb induced neurotoxicity in chickens. These findings are supported by another study, which observed that Andean condors under Pb stress showed reduced activity and lethargy [30]. Additionally, we observed that Se supplement relieved the above neurological symptoms in chickens, which indicated that Se mitigated Pb-caused neurotoxicity. Microstructural observation is a useful tool for exploring organ injury in animals [31,32]. Previous studies reported that Pb caused inflammatory injury in rat Ces [33] and chicken testis [34] by microstructural observation. Therefore, in order to investigate whether Pb can cause damage to the chicken nervous system, we also observed the microstructures of chicken Ces and Thi. Our experiment revealed that Pb exposure led to damage in both chicken Ces and Thi at three time points. The signs of cerebellar injury included increased nerve fiber space, the presence of red blood cells, and inflammatory cell infiltration. The signs of thalamic injury included increased nerve fiber space, the presence of red blood cells, decreased Nissl bodies, and hydropic degeneration. Other reports documented similar phenomena. Li et al. (2021) found that heavy metal cobalt induced an increase in nerve fiber space, a decrease in Nissl bodies, and caused inflammatory damage in the mouse hippocampus [35]. Pb exposure resulted in hydropic degeneration and inflammatory injury in the rat cerebral cortex [36]. Moreover, we found that the degree of damage in chicken Ces and Thi, as well as the IBR values of selenoproteins, oxidative stress indexes, and inflammatory-related factors, increased in a time-dependent manner in the Pb-exposed group, indicating that Pb time-dependently injured chicken Ces and Thi. Interestingly, on day 90, Thi showed more severe injury than Ces under Pb stress, as evidenced by the observation of a large amount of hydrodynamic generation in Thi, whereas this was not observed in Ces. The IBR values of oxidative stress indexes and inflammatory-related factors were higher in Thi than those in Ces, which supported the findings of microstructural observation and required further study. Additionally, Se could reduce Pb toxicity. Thus, we further observed the microstructures of chicken Ces and Thi under the condition of Pb and Se co-treatment. The results showed that Se alleviated Pb-caused inflammatory damage in both chicken Ces and Thi. Two studies support our findings in which inflammatory cell infiltration, hydropic degeneration, and inflammatory injury caused by chronic unpredictable mild stress in rat hippocampus [37], as well as caused by excess Pb in chicken testes [34] were relieved by Se supplementation. 

A variety of inflammatory-related factors are involved in the molecular mechanism of inflammatory damage. *IL-2*, a T-cell growth factor, exhibits an anti-inflammatory effect. *IL-4* acts as an early promoter of inflammation. *IL-6* can promote the development of inflammation. *IL-12β* is an important pro-inflammatory cytokine. *IL-17* can enhance the inflammatory response in organisms. *INF-γ* plays a vital role in host defense and can be reduced in the presence of inflammation. NO serves as a pro-inflammatory medium. In this study, we measured the above seven inflammatory-related factors. The results showed a decrease in *IL-2* and *INF-γ* and, on the contrary, an increase in *IL-4*, *IL-6*, *IL-12β*, *IL-17*, and NO, which meant that Pb induced an inflammatory response and demonstrated that Pb led to inflammatory injury in chicken Ces and Thi. Other studies are consistent with our findings. Han et al. (2020) found that exposure to excess ammonia gas reduced *IL-2* and *INF-γ*, elevated *IL-6* and *IL-17*, and resulted in inflammatory injury in chicken kidneys [38]. Up-regulated *IL-4* and *IL-6*, down-regulated *INF-γ*, and inflammatory damage were found in the small intestines of Cd-exposed pigs [11]. In addition, previous studies found that mice with *IL-2* deletion had elevated serum *IL-17* concentration [39]. *IL-17* treatment increased NO production, and *IL-17*-specific neutralizing antibodies decreased NO production in mouse insulinoma cell line MIN6 [40]. Herein, our findings also indicated that the *IL-2*/*IL-17*–NO pathway participated in the molecular mechanism of Pb-caused inflammatory damage in chicken Ces and Thi. This is the first study to clarify the *IL-2*/*IL-17*–NO pathway in the context of poisoning. 

Several studies reported that Se moderated Pb-induced inflammation in chickens. Se relived an increase in NO and inflammatory damage in the livers of Pb-treated chickens [41]. Liu et al. (2019) recorded that Se had a mitigative effect on Pb-caused *IL-4* and NO elevation and inflammatory injury in chicken skeletal muscles [42]. Under the Pb exposure condition, the up-regulation of *IL-6* and NO, the down-regulation of *INF-γ*, and inflammatory damage in chicken testes were relieved by dietary Se supplementation [43]. We also found that the supplementation of Se in the chicken diet antagonized the effect of Pb on all the detected seven inflammatory-related factors and alleviated Pb-caused inflammatory damage in chicken Ces and Thi, further demonstrating the mitigation effect of Se on inflammation caused by Pb in chickens and indicating that the *IL-2*/*IL-17*–NO pathway was involved in the molecular mechanism of Se antagonizing Pb toxicity in chickens.

Researchers discovered that environmental pollutants caused oxidative stress in animals [44,45,46]. Li et al. (2022) mentioned that both oxidative stress and inflammatory damage occurred in mouse hearts under doxorubicin treatment [47]. Chen et al. (2024b) exhibited that excess ammonia induced oxidative stress and inflammatory response in chicken splenic lymphocytes [48]. Thereby, we explored whether oxidative stress was involved in the molecular mechanism of Pb poisoning. MDA is an oxidation product in organisms and is harmful to organisms. GSH, which can scavenge oxidants, is considered to be the first line of defense against oxidative damage in cells. TAC is a reflection of the overall antioxidant capacity in organs. Hence, we measured MDA, GSH, and TAC and found that Pb exposure elevated MDA and, on the contrary, reduced GSH and TAC at three time points both in chicken Ces and Thi, meaning that Pb resulted in oxidative stress in chicken Ces and Thi. Three reports are in concordance with our findings. Oxidative stress occurred with increased MDA and decreased GSH in the livers of chlorpyrifos-treated common carps [49], as well as in the cerebral cortex of Pb-treated rats [36]. Pancreatic oxidative stress occurred in bisphenol A-exposed chickens with up-regulated MDA and down-regulated GSH and TAC [8]. What is more, GSH inhibitor R-sulfoximine blocked *IL-2* secretion in mouse T cells [50]. Thus, our findings also indicated that oxidative stress mediated the inflammatory injury caused by Pb via the GSH–*IL-2* axis in chicken Ces and Thi. Additionally, in our experiment, increased MDA and decreased GSH and TAC were reversed by dietary Se, which demonstrated that Se relieved Pb-induced oxidative stress in chicken Ces and Thi. Similarly, Se supplementation mitigated Cd-caused GSH decrease and oxidative stress in chicken hepatocytes [51], as well as Pb-caused MDA increase and oxidative stress in chicken testes [43]. 

Both oxidative stress and selenoprotein decrease can occur in the case of heavy metal exposure [21,52]. We also explored whether selenoproteins took part in the molecular mechanism of Pb poisoning both in chicken Ces and Thi. Selenoproteins (such as *SPS2*, GPxs, Dios, *SelH*, *SelI*, *SelK*, *SelM*, *SelO*, *SelS*, *SelT*, *SelU*, *SelP1*, *SelPb*, *Sepn1*, *Sepw1*, *Sepx1*, and *Sep15*) are a class of Se-containing proteins, which are important biomolecules for Se to achieve physiological functions. *SPS2* is involved in the synthesis of other selenoproteins. *GPxs* can remove harmful metabolites from organisms. *Dios* regulate thyroid hormone activity, which realizes the metabolic regulation ability of Se. *SelH*, *SelK*, *SelM*, *SelO*, *SelT*, *SelU*, Selw1, *Sepx1*, and *Sep15* can maintain the oxidation and reduction steady state. *SelS* participates in the degradation of misfolded proteins, *SelI* takes part in phospholipid biosynthesis, and SelP partakes in the transport of Se. In our experiment, mRNA expressions of *SPS2*, *GPx1-4*, *Dio1-3*, *SelH*, *SelI*, *SelK*, *SelM*, *SelO*, *SelS*, *SelT*, *SelU*, *SelP1*, *SelPb*, *Sepn1*, *Sepw1*, *Sepx1*, and *Sep15* were down-regulated under Pb exposure, which indicated that Pb caused selenoprotein suppression in chicken Ces and Thi. Similar to our findings, Pb exposure inhibited *GPx1*, *GPx4*, *SelK*, and *Sep15* in mouse livers [21]. Cd reduced ten selenoproteins (*GPx1-4*, *Dio3*, *SelK*, *SelO*, *SelP1*, *SelT*, and Sepw) in chicken cerebrums [52]. Moreover, researchers found that *GPx1* was reduced in NIH3T3 cells in which *SPS2* was knocked down [53], and *SelH*, *SelP1*, and *Sepw1* were reduced in chicken myoblasts in which *SelK* was knocked down [54]. Hence, our results indicated the involvement of the *SPS2*–*GPx1*, *SelK*–*SelH*, *SelK*–*SelP1*, and *SelK*–*Sepw1* axes in Pb-caused selenoprotein suppression in chicken Ces and Thi. Additionally, *Dio2* treatment attenuated doxorubicin-induced oxidative stress in C2C12 cells [55]. Oxidative stress occurred in *SelK*-deficient mouse C2C12 myoblasts [56]. Tran et al. (2017) reported that *GPx1* knockout and *GPx1* overexpression down-regulated and up-regulated GSH, respectively, in the mouse prefrontal cortex [57]. Thus, our findings indicated that selenoprotein suppression mediated oxidative stress via the *SPS2*–*GPx1*–GSH pathway and oxidative stress took part in selenoprotein suppression-mediated inflammatory damage through the *SPS2*–*GPx1*–GSH–*IL-2*/*IL-17*–NO pathway in the Ces and Thi of Pb-exposed chickens. We found for the first time that the *SPS2*–*GPx1*–GSH–*IL-2*/*IL-17*–NO pathway participated in the molecular mechanism of poisoning. However, the *SPS2*–*GPx1*–GSH–*IL-2*/*IL-17*–NO pathway was not verified under an in vitro Pb/Se cell model. More in-depth studies are needed in the future. In addition, we found that Pb time-dependently decreased *SPS2* in chicken Ces, as well as time-dependently decreased *SelH*, *SelU*, *SelO*, and *SelPb* in chicken Thi, which also requires further study. Interestingly, the IBR values of selenoproteins in Ces were higher than those in Thi at all three time points under Pb stress, which suggested that selenoproteins in Ces were more sensitive to Pb exposure than those in Thi.

In our experiment, Se supplementation up-regulated Pb-inhibited *SPS2*, *GPx1-4*, *Dio1-3*, *SelH*, *SelI*, *SelK*, *SelM*, *SelO*, *SelS*, *SelT*, *SelU*, *SelP1*, *SelPb*, *Sepn1*, *Sepw1*, *Sepx1*, and *Sep15*, which suggested that Se antagonized selenoprotein suppression-mediated oxidative stress caused by Pb in chicken Ces and Thi. Similar to our findings, Zhang et al. (2020) confirmed that Se relieved the Cd-caused decrease in GSH and thirteen selenoproteins (*GPx1*, *GPx3*, *GPx4*, *SelI*, *SelK*, *SelM*, *SelO*, *SelS*, *SelT*, *Sepw1*, *SelP1*, *Sepx1*, and *Sep15*) and oxidative stress in chicken hepatocytes [51]. Pb-inhibited *GPx1-4*, *Dio1*-3, *SelH*, *SelI*, *SelK*, *SelM*, *SelO*, *SelS*, *SelT*, *SelU*, *SelP1*, *SelPb*, *Sepn1*, *Sepw1*, *Sepx1*, *Sep15*, and *SPS2* were increased by Se in chicken brainstems [22]. Se has anti-intoxicating and anti-stress properties, which are a wide concern for humans. In our study, Se supplementation alleviated Pb-caused neurotoxicity in chickens. Other studies also found that Se alleviated toxic environmental pollutant-caused toxicity and high temperature-caused heat stress in chickens. Se relieved kidney injury caused by heavy metals (such as Cd [58] and chromium [59]), splenic injury caused by ammonia gas [60], brain injury caused by decabromodiphenyl ether [61], and liver damage caused by high temperature [62]. Eid et al. (2023) found that insecticide imidacloprid-decreased growth performance was reversed by Se [63]. Thus, it is recommended to supplement Se appropriately in breeding practice, especially in areas with environmental pollution and extreme environments.

## 5. Conclusions

In conclusion, Pb exposure led to inflammatory damage in both chicken Ces and Thi. Moreover, excess Pb down-regulated *IL-2* and *INF-γ* and, on the contrary, up-regulated *IL-4*, *IL-6*, *IL-12β*, *IL-17*, and NO, indicating that the *IL-2*/*IL-17*–NO pathway was involved in the molecular mechanism of Pb-induced inflammatory injury in chicken Ces and Thi. Furthermore, Pb treatment resulted in a decrease in twenty-two selenoproteins (*GPx1-4*, *Dio1*-3, *SelH*, *SelI*, *SelK*, *SelM*, *SelO*, *SelS*, *SelT*, *SelU*, *SelP1*, *SelPb*, *Sepn1*, *Sepw1*, *Sepx1*, *Sep15*, and *SPS2*) and two anti-oxidant indexes (GSH and TAC), an increase in MDA, and the occurrence of oxidative stress. Pb-inhibited selenoprotein-mediated oxidative stress via the *SPS2*–*GPx1*–GSH pathway, and oxidative stress-mediated inflammatory response via the GSH–*IL-2* axis, finally led to inflammatory injury with the involvement of the *SPS2*–*GPx1*–GSH–*IL-2*/*IL-17*–NO pathway. Interestingly, a time-dependent manner was found on *SPS2* in Ces, as well as *SelH*, *SelU*, SepO, and *SelPb* in Thi, under the Pb exposure condition. Additionally, dietary Se alleviated all the above changes caused by Pb, which meant that Se relieved selenoprotein inhibition and oxidative stress-mediated inflammatory injury in the Ces and Thi of Pb-treated chickens. Our study provided new insights into the complex molecular mechanism of the Pb-Se interaction and also revealed the detoxification effect and detoxification mechanism of Se.

## Figures and Tables

**Table 1:** Primer sequences of all genes detected in our experiment.

Genes	Serial Numbers	Primer Sequences	
*β-actin*	L08165	F: 5’-CCGCTCTATGAAGGCTACGC-3’	R: 5’-CTC TCG GCT GTG GTGGTG AA-3’
*GPx1*	NM_001277853.1	F: 5’-ACGGCGCATCTTCCAAAG-3’	R: 5’-TGTTCCCCCAACCATTTCTC-3’
*GPx2*	NM_001277854.1	F: 5’-ATCGCCAAGTCCTTCTACGA-3’	R: 5’-ACGTTCTCGATGAGGACCAC-3’
*GPx3*	NM_001163232.2	F: 5’-CCTGCAGTACCTCGAACTGA-3’	R: 5’-CTTCAGTGCAGGGAGGATCT-3’
*GPx4*	AF498316.2	F: 5’-CTTCGTCTGCATCATCACCAA-3’	R: 5’-TCGACGAGCTGAGTGTAATTCAC-3’
*Dio1*	NM_001097614.1	F: 5’-GCGCTATACCACAGGCAGTA-3’	R: 5’-GGTCTTGCAAATGTCACCAC-3’
*Dio2*	NM_204114.3	F: 5’-ATTTGCTGATCACGCTTCAG-3’	R: 5’-GCTCAGAAACAGCACCATGT-3’
*Dio3*	NM_001122648.1	F: 5’-CTGTGCATTCGCAAGAAGAT-3’	R: 5’-GCCGACTTGAAGAAGTCCAG-3’
*SelH*	NM_001277865.1	F: 5’- CATCGAGCACTGCCGTAG-3’	R: 5’- GACACCTCGAAGCTGTTCCT-3’
*SelI*	NM_001031528.2	F: 5’-TGCCAGCCTCTGAACTGGAT-3’	R: 5’-TGCAAACCCAGACATCACCAT-3’
*SelK*	NM_001025441.2	F: 5’- ATGACGACCACCCTCACGAT-3’	R: 5’- CCAGCGTTAACCGGAATGAT-3
*SelM*	NM_001277859.1	F: 5’-AAGAAGGACCACCCAGACCT-3’	R: 5’-GCTGTCCTGTCTCCCTCATC-3’
*SelO*	NM_001115017.1	F: 5’-CCAGCGTTAACCGGAATGAT-3’	R: 5’-GCCTACAGAATGGATCCAACTGA-3’
*SelS*	NM_173120.2	F: 5’-GCCTGCGTCGCCATCTATCTCA-3’	R: 5’-TTCTGCCTTCGCTTCTGTTCTTCAA-3’
*SelT*	NM_001006557.3	F: 5’-AGGAGTACATGCGGGTCATCA-3’	R: 5’-GACAGACAGGAAGGATGCTATGTG-3’
*SelU*	NM_001193518.1	F: 5’-GATGCTTTCAGGCTTCTTCC-3’	R: 5’-CTGTCTTCCTGCTCCAATCA-3’
*SelPb*	XM_003641687.2	F: 5’-AGGCCAACAGTACCATGGAG-3’	R: 5’-GTGGTGAGGATGGAGATGGT-3’
*Sepn1*	NM_001114972.1	F: 5’-CAGGATCCATGCTGAGTTCCA-3’	R: 5’-GAGAGGACGATGTAACCCGTAAAC-3’
*SelP1*	NM_001031609	F: 5’-CCAAGTGGTCAGCATTCACATC-3’	R: 5’-ATGACGACCACCCTCACGAT-3’
*Sepx1*	NM_001135558.2	F: 5’-TGGCAAGTGTGGCAATGG-3’	R: 5’-GAATTTGAGCGAGCTGCTGAAT-3’
*Sepw1*	NM_001166327.1	F: 5’-TGGTGTGGGTCTGCTTTACG-3’	R: 5’-CCAAAGCTGGAAGGTGCAA-3’
*Sep15*	NM_001012926.2	F: 5’-ACTTGGCTTCTCCAGTAACTTGCT-3’	R: 5’-GCCTACAGAATGGATCCAACTGA-3’
*SPS2*	BM489698.1	F: 5’-CGTTGGGTATCGGAACTGAC-3’	R: 5’-CGTCCACCAGAGGGTAGAAA-3’
*IL-2*	AY510091	F:5′-GAACCTCAAGAGTCTTACGGGTCTA-3′	R: 5′-ACAAAGTTGGTCAGTTCATGGAGA-3′
*IL-4*	AJ621249	F: 5′-GTGCCCACGCTGTGCTTAC-3′	R: 5′-AGGAAACCTCTCCCTGGATGTC-3′
*IL-6*	NM204628	F: 5′-AAATCCCTCCTCGCCAATCT-3′	R: 5′-CCCTCACGGTCTTCTCCATAAA-3′
*IL-12β*	AJ564201.1	F: 5′-TGTCTCACCTGCTATTTGCCTTAC-3′	R:5′-CATACACATTCTCTCTAAGTTTCCACTGT-3′
*IL-17*	AY744450	F: 5′-CATGTTGTCAGCCAGCATTTCT-3′	R: 5′-CATCTTTTTGGGTTAGGCATCC-3′
*INF-* *γ*	DQ470471	F: 5′-AAGTCATAGCGGCACATCAAAC-3′	R: 5′-CTGGAATCTCATGTCGTTCATCG-3′

**Table 2:** The mRNA levels of selenoproteins in chicken Ces and Thi. Different lowercase letters indicate that there are significant (*p* < 0.05) differences among different groups at the same time point, and different capital letters indicate that there are significant (*p* < 0.05) differences among different time points in the same group. Each value is shown as mean ± standard deviation (*n* = 5).

Organs	Items	Days	The Control Group	The Se Group	The Pb Group	The Se/Pb Group
Cerebellum	*GPx1*	30 d	0.98 ± 0.04 ^Aa^	1.05 ± 0.04 ^Aa^	0.62 ± 0.05 ^Ab^	0.83 ± 0.01 ^Ac^
		60 d	1.01 ± 0.01 ^Aa^	0.97 ± 0.04 ^Aa^	0.45 ± 0.11 ^ABb^	0.72 ± 0.02 ^Bc^
		90 d	1.05 ± 0.05 ^Aa^	1.00 ± 0.04 ^Aa^	0.31 ± 0.01 ^Bb^	0.72 ± 0.02 ^Bc^
	*GPx2*	30 d	1.00 ± 0.03 ^Aa^	0.98 ± 0.02 ^Aa^	0.55 ± 0.05 ^Ab^	0.65 ± 0.02 ^Ac^
		60 d	1.01 ± 0.02 ^Aa^	1.03 ± 0.02 ^Aa^	0.41 ± 0.04 ^Bb^	0.68 ± 0.06 ^Ac^
		90 d	1.04 ± 0.06 ^Aa^	0.98 ± 0.03 ^Aa^	0.43 ± 0.01 ^Bb^	0.67 ± 0.02 ^Ac^
	*GPx3*	30 d	0.97 ± 0.02 ^Aa^	1.01 ± 0.04 ^Aa^	0.72 ± 0.07 ^Ab^	0.94 ± 0.05 ^Aa^
		60 d	1.03 ± 0.02 ^Aa^	1.02 ± 0.02 ^Aa^	0.68 ± 0.06 ^Ab^	0.88 ± 0.02 ^Ac^
		90 d	1.00 ± 0.08 ^Aa^	1.04 ± 0.01 ^Aa^	0.64 ± 0.05 ^Ab^	0.77 ± 0.05 ^Ac^
	*GPx4*	30 d	1.00 ± 0.02 ^Aa^	1.07 ± 0.03 ^Aa^	0.79 ± 0.02 ^Ab^	0.80 ± 0.06 ^Ab^
		60 d	1.02 ± 0.06 ^Aa^	1.03 ± 0.01 ^Aa^	0.67 ± 0.03 ^Bb^	0.78 ± 0.02 ^Ac^
		90 d	1.02 ± 0.01 ^Aa^	1.07 ± 0.02 ^Aa^	0.69 ± 0.04 ^Bb^	0.80 ± 0.05 ^Ac^
	*Dio1*	30 d	0.99 ± 0.03 ^Aa^	1.02 ± 0.02 ^Aa^	0.27 ± 0.03 ^Ab^	0.71 ± 0.06 ^Ac^
		60 d	0.98 ± 0.02 ^Aa^	1.00 ± 0.02 ^Aa^	0.26 ± 0.02 ^Ab^	0.75 ± 0.02 ^Ac^
		90 d	1.02 ± 0.03 ^Aa^	1.04 ± 0.02 ^Aa^	0.26 ± 0.01 ^Ab^	0.66 ± 0.04 ^Ac^
	*Dio2*	30 d	1.01 ± 0.02 ^Aa^	1.04 ± 0.05 ^Aa^	0.42 ± 0.06 ^Ab^	0.74 ± 0.03 ^Ac^
		60 d	0.99 ± 0.02 ^Aa^	0.98 ± 0.04 ^Aa^	0.35 ± 0.06 ^Ab^	0.74 ± 0.08 ^Ac^
		90 d	1.02 ± 0.03 ^Aa^	1.04 ± 0.03 ^Aa^	0.39 ± 0.07 ^Ab^	0.73 ± 0.08 ^Ac^
	*Dio3*	30 d	0.97 ± 0.05 ^Aa^	1.07 ± 0.02 ^Aa^	0.41 ± 0.07 ^Ab^	0.81 ± 0.05 ^Ac^
		60 d	0.96 ± 0.05 ^Aa^	1.00 ± 0.06 ^Aa^	0.36 ± 0.06 ^Ab^	0.76 ± 0.05 ^Ac^
		90 d	1.03 ± 0.01 ^Aa^	1.02 ± 0.04 ^Aa^	0.37 ± 0.01 ^Ab^	0.75 ± 0.03 ^Ac^
	*SelH*	30 d	0.99 ± 0.03 ^Aa^	1.02 ± 0.05 ^Aa^	0.64 ± 0.01 ^Ab^	0.72 ± 0.06 ^Ac^
		60 d	1.01 ± 0.02 ^Aa^	0.98 ± 0.09 ^Aa^	0.61 ± 0.01 ^Ab^	0.73 ± 0.03 ^Ac^
		90 d	1.01 ± 0.06 ^Aa^	1.05 ± 0.03 ^Aa^	0.60 ± 0.03 ^Ab^	0.72 ± 0.02 ^Ac^
	*SelI*	30 d	1.02 ± 0.01 ^Aa^	1.04 ± 0.03 ^Aa^	0.51 ± 0.07 ^Ab^	0.85 ± 0.01 ^Ac^
		60 d	0.99 ± 0.06 ^Aa^	1.01 ± 0.05 ^Aa^	0.48 ± 0.02 ^Ab^	0.81 ± 0.05 ^Ac^
		90 d	1.01 ± 0.03 ^Aa^	1.05 ± 0.05 ^Aa^	0.46 ± 0.02 ^Bb^	0.82 ± 0.08 ^Ac^
	*SelK*	30 d	0.98 ± 0.02 ^Aa^	1.05 ± 0.04 ^Aa^	0.64 ± 0.03 ^Ab^	0.85 ± 0.04 ^Ac^
		60 d	1.01 ± 0.09 ^Aa^	0.99 ± 0.05 ^Aa^	0.65 ± 0.03 ^Ab^	0.81 ± 0.03 ^Ac^
		90 d	1.04 ± 0.06 ^Aa^	1.06 ± 0.01 ^Aa^	0.66 ± 0.03 ^Ab^	0.78 ± 0.01 ^Ac^
	*SelM*	30 d	1.00 ± 0.04 ^Aa^	1.04 ± 0.01 ^Aa^	0.54 ± 0.09 ^Ab^	0.83 ± 0.04 ^Ac^
		60 d	0.98 ± 0.04 ^Aa^	1.03 ± 0.01 ^Aa^	0.50 ± 0.02 ^Ab^	0.83 ± 0.05 ^Ac^
		90 d	1.04 ± 0.04 ^Aa^	1.06 ± 0.02 ^Aa^	0.49 ± 0.03 ^Ab^	0.84 ± 0.02 ^Ac^
	*SelO*	30 d	1.01 ± 0.03 ^Aa^	1.04 ± 0.02 ^Aa^	0.62 ± 0.05 ^Ab^	0.85 ± 0.04 ^Ac^
		60 d	0.99 ± 0.05 ^Aa^	1.03 ± 0.05 ^Aa^	0.65 ± 0.05 ^Ab^	0.83 ± 0.03 ^Ac^
		90 d	1.05 ± 0.04 ^Aa^	1.01 ± 0.05 ^Aa^	0.56 ± 0.04 ^Ab^	0.80 ± 0.01 ^Ac^
	*SelS*	30 d	1.01 ± 0.04 ^Aa^	1.01 ± 0.06 ^Aa^	0.45 ± 0.02 ^Ab^	0.85 ± 0.05 ^Ac^
		60 d	1.00 ± 0.02 ^Aa^	1.02 ± 0.01 ^Aa^	0.43 ± 0.05 ^Ab^	0.86 ± 0.20 ^Ac^
		90 d	1.01 ± 0.05 ^Aa^	1.01 ± 0.05 ^Aa^	0.40 ± 0.08 ^Ab^	0.84 ± 0.04 ^Ac^
	*SelT*	30 d	1.00 ± 0.03 ^Aa^	1.02 ± 0.01 ^Aa^	0.61 ± 0.03 ^Ab^	0.82 ± 0.07 ^Ac^
		60 d	0.99 ± 0.04 ^Aa^	1.04 ± 0.05 ^Aa^	0.56 ± 0.05 ^Ab^	0.82 ± 0.02 ^Ac^
		90 d	1.03 ± 0.05 ^Aa^	0.98 ± 0.03 ^Aa^	0.55 ± 0.05 ^Ab^	0.70 ± 0.06 ^Ac^
	*SelU*	30 d	0.97 ± 0.06 ^Aa^	0.99 ± 0.06 ^Aa^	0.61 ± 0.07 ^Ab^	0.79 ± 0.04 ^Ac^
		60 d	0.99 ± 0.06 ^Aa^	1.03 ± 0.05 ^Aa^	0.56 ± 0.02 ^Ab^	0.72 ± 0.03 ^ABc^
		90 d	1.00 ± 0.06 ^Aa^	1.04 ± 0.02 ^Aa^	0.51 ± 0.04 ^Ab^	0.67 ± 0.03 ^Bc^
	*SelPb*	30 d	0.96 ± 0.02 ^Aa^	1.08 ± 0.02 ^Ab^	0.60 ± 0.06 ^Ac^	0.70 ± 0.03 ^Ad^
		60 d	0.97 ± 0.05 ^Aa^	1.06 ± 0.03 ^Aa^	0.62 ± 0.05 ^Ab^	0.72 ± 0.02 ^Ac^
		90 d	1.00 ± 0.03 ^Aa^	1.06 ± 0.01 ^Aa^	0.58 ± 0.04 ^Ab^	0.76 ± 0.02 ^Ac^
	*Sepn1*	30 d	0.98 ± 0.04 ^Aa^	0.99 ± 0.03 ^Aa^	0.49 ± 0.05 ^Ab^	0.84 ± 0.01 ^Ac^
		60 d	1.00 ± 0.01 ^Aa^	0.99 ± 0.02 ^Aa^	0.43 ± 0.05 ^Ab^	0.84 ± 0.03 ^Ac^
		90 d	0.99 ± 0.01 ^Aa^	1.03 ± 0.01 ^Aa^	0.42 ± 0.07 ^Ab^	0.85 ± 0.02 ^Ac^
	*SelP1*	30 d	1.01 ± 0.03 ^Aa^	1.02 ± 0.03 ^Aa^	0.54 ± 0.02 ^Ab^	0.77 ± 0.02 ^Ac^
		60 d	0.96 ± 0.10 ^Aa^	1.05 ± 0.02 ^Aa^	0.50 ± 0.07 ^Ab^	0.74 ± 0.04 ^Ac^
		90 d	1.01 ± 0.03 ^Aa^	1.04 ± 0.03 ^Aa^	0.46 ± 0.02 ^Ab^	0.66 ± 0.04 ^Bc^
	*Sepx1*	30 d	1.02 ± 0.05 ^Aa^	1.07 ± 0.02 ^Aa^	0.73 ± 0.08 ^Ab^	0.79 ± 0.05 ^Ab^
		60 d	0.98 ± 0.02 ^Aa^	1.03 ± 0.05 ^Aa^	0.53 ± 0.02 ^Ab^	0.79 ± 0.03 ^Ac^
		90 d	1.03 ± 0.04 ^Aa^	1.05 ± 0.03 ^Aa^	0.44 ± 0.04 ^Bb^	0.83 ± 0.05 ^Ac^
	*Sepw1*	30 d	0.98 ± 0.03 ^Aa^	1.04 ± 0.03 ^Aa^	0.74 ± 0.04 ^Ab^	0.86 ± 0.02 ^Ac^
		60 d	1.06 ± 0.05 ^Aa^	1.07 ± 0.02 ^Aa^	0.63 ± 0.03 ^Bb^	0.88 ± 0.07 ^Ac^
		90 d	1.01 ± 0.02 ^Aa^	1.03 ± 0.05 ^Aa^	0.54 ± 0.04 ^Bb^	0.90 ± 0.09 ^Ac^
	*Sep15*	30 d	1.00 ± 0.04 ^Aa^	1.00 ± 0.05 ^Aa^	0.47 ± 0.03 ^Ab^	0.71 ± 0.04 ^Ac^
		60 d	1.02 ± 0.02 ^Aa^	1.06 ± 0.01 ^Aa^	0.32 ± 0.03 ^Bb^	0.68 ± 0.03 ^Ac^
		90 d	1.01 ± 0.02 ^Aa^	1.02 ± 0.07 ^Aa^	0.27 ± 0.04 ^Bb^	0.63 ± 0.07 ^Ac^
	*SPS2*	30 d	1.00 ± 0.03 ^Aa^	0.99 ± 0.04 ^Aa^	0.75 ± 0.03 ^Ab^	0.86 ± 0.05 ^Ac^
		60 d	1.06 ± 0.03 ^Aa^	1.05 ± 0.03 ^Aa^	0.66 ± 0.02 ^Bb^	0.80 ± 0.03 ^ABc^
		90 d	1.04 ± 0.04 ^Aa^	1.00 ± 0.01 ^Aa^	0.46 ± 0.03 ^Cb^	0.73 ± 0.01 ^Bc^
Thalamus	*GPx1*	30 d	0.98 ± 0.02 ^Aa^	1.03 ± 0.02 ^Aa^	0.68 ± 0.04 ^Ab^	0.82 ± 0.03 ^Ac^
		60 d	1.05 ± 0.03 ^Aa^	1.04 ± 0.01 ^Aa^	0.65 ± 0.01 ^Ab^	0.87 ± 0.02 ^Ac^
		90 d	1.00 ± 0.03 ^Aa^	1.04 ± 0.04 ^Aa^	0.48 ± 0.05 ^Ab^	0.86 ± 0.02 ^Ac^
	*GPx2*	30 d	1.01 ± 0.04 ^Aa^	1.05 ± 0.02 ^Aa^	0.61 ± 0.04 ^Ab^	0.80 ± 0.02 ^Ac^
		60 d	1.05 ± 0.04 ^Aa^	1.06 ± 0.03 ^Aa^	0.57 ± 0.09 ^Ab^	0.81 ± 0.03 ^Ac^
		90 d	1.03 ± 0.03 ^Aa^	1.05 ± 0.02 ^Aa^	0.55 ± 0.04 ^Ab^	0.83 ± 0.03 ^Ac^
	*GPx3*	30 d	0.99 ± 0.03 ^Aa^	1.03 ± 0.02 ^Aa^	0.74 ± 0.08 ^Ab^	0.81 ± 0.03 ^Ac^
		60 d	1.01 ± 0.02 ^Aa^	1.03 ± 0.01 ^Aa^	0.76 ± 0.03 ^Ab^	0.85 ± 0.08 ^Ac^
		90 d	1.03 ± 0.02 ^Aa^	1.01 ± 0.04 ^Aa^	0.77 ± 0.08 ^Ab^	0.83 ± 0.04 ^Ac^
	*GPx4*	30 d	1.03 ± 0.05 ^Aa^	1.05 ± 0.04 ^Aa^	0.52 ± 0.08 ^Ab^	0.85 ± 0.02 ^Ac^
		60 d	1.03 ± 0.01 ^Aa^	1.03 ± 0.01 ^Aa^	0.73 ± 0.03 ^Ab^	0.85 ± 0.03 ^Ac^
		90 d	1.02 ± 0.04 ^Aa^	1.02 ± 0.02 ^Aa^	0.72 ± 0.04 ^Ab^	0.81 ± 0.02 ^Ac^
	*Dio1*	30 d	0.97 ± 0.02 ^Aa^	1.02 ± 0.04 ^Aa^	0.38 ± 0.04 ^Ab^	0.85 ± 0.01 ^Ac^
		60 d	1.03 ± 0.03 ^Aa^	1.06 ± 0.03 ^Aa^	0.34 ± 0.02 ^Ab^	0.85 ± 0.03 ^Ac^
		90 d	1.00 ± 0.03 ^Aa^	1.04 ± 0.03 ^Aa^	0.34 ± 0.01 ^Ab^	0.87 ± 0.03 ^Ac^
	*Dio2*	30 d	1.00 ± 0.06 ^Aa^	1.07 ± 0.02 ^Aa^	0.59 ± 0.05 ^Ab^	0.90 ± 0.03 ^Ac^
		60 d	1.04 ± 0.03 ^Aa^	1.06 ± 0.02 ^Aa^	0.51 ± 0.04 ^ABb^	0.92 ± 0.04 ^Ac^
		90 d	1.01 ± 0.03 ^Aa^	1.04 ± 0.01 ^Aa^	0.47 ± 0.02 ^Bb^	0.95 ± 0.02 ^Ac^
	*Dio3*	30 d	1.02 ± 0.01 ^Aa^	1.06 ± 0.02 ^Aa^	0.55 ± 0.07 ^Ab^	0.83 ± 0.05 ^Ac^
		60 d	1.04 ± 0.04 ^Aa^	1.02 ± 0.08 ^Aa^	0.47 ± 0.04 ^ABb^	0.76 ± 0.06 ^Ac^
		90 d	1.04 ± 0.03 ^Aa^	1.01 ± 0.03 ^Aa^	0.36 ± 0.01 ^Bb^	0.76 ± 0.06 ^Ac^
	*SelH*	30 d	0.99 ± 0.03 ^Aa^	1.06 ± 0.03 ^Aa^	0.56 ± 0.03 ^Ab^	0.91 ± 0.05 ^Ac^
		60 d	1.03 ± 0.04 ^Aa^	1.04 ± 0.01 ^Aa^	0.46 ± 0.03 ^Bb^	0.87 ± 0.06 ^Ac^
		90 d	1.01 ± 0.02 ^Aa^	1.04 ± 0.02 ^Aa^	0.36 ± 0.03 ^Cb^	0.85 ± 0.02 ^Ac^
	*SelI*	30 d	1.00 ± 0.02 ^Aa^	1.03 ± 0.03 ^Aa^	0.37 ± 0.03 ^Ab^	0.96 ± 0.03 ^Ac^
		60 d	1.03 ± 0.02 ^Aa^	1.04 ± 0.03 ^Aa^	0.36 ± 0.02 ^Ab^	0.88 ± 0.05 ^Ac^
		90 d	1.04 ± 0.04 ^Aa^	1.00 ± 0.02 ^Aa^	0.30 ± 0.05 ^Ab^	0.88 ± 0.06 ^Ac^
	*SelK*	30 d	0.98 ± 0.06 ^Aa^	1.05 ± 0.02 ^Aa^	0.47 ± 0.04 ^Ab^	0.87 ± 0.04 ^Ac^
		60 d	1.02 ± 0.03 ^Aa^	1.04 ± 0.04 ^Aa^	0.48 ± 0.03 ^Ab^	0.85 ± 0.02 ^Ac^
		90 d	0.98 ± 0.03 ^Aa^	1.06 ± 0.03 ^Aa^	0.46 ± 0.03 ^Ab^	0.87 ± 0.02 ^Ac^
	*SelM*	30 d	0.98 ± 0.05 ^Aa^	1.06 ± 0.04 ^Aa^	0.54 ± 0.01 ^Ab^	0.89 ± 0.02 ^Ac^
		60 d	1.04 ± 0.03 ^Aa^	1.05 ± 0.02 ^Aa^	0.34 ± 0.04 ^Bb^	0.87 ± 0.04 ^ABc^
		90 d	1.03 ± 0.03 ^Aa^	1.05 ± 0.05 ^Aa^	0.36 ± 0.03 ^Bb^	0.75 ± 0.08 ^Bc^
	*SelO*	30 d	0.99 ± 0.04 ^Aa^	1.05 ± 0.03 ^Aa^	0.50 ± 0.02 ^Ab^	0.75 ± 0.02 ^Ac^
		60 d	0.99 ± 0.03 ^Aa^	1.03 ± 0.02 ^Aa^	0.44 ± 0.02 ^Bb^	0.73 ± 0.03 ^Ac^
		90 d	1.04 ± 0.05 ^Aa^	1.03 ± 0.02 ^Aa^	0.37 ± 0.02 ^Cb^	0.77 ± 0.02 ^Ac^
	*SelS*	30 d	1.02 ± 0.02 ^Aa^	1.04 ± 0.01 ^Aa^	0.51 ± 0.06 ^Ab^	0.85 ± 0.02 ^Ac^
		60 d	1.01 ± 0.03 ^Aa^	1.05 ± 0.01 ^Aa^	0.34 ± 0.09 ^Bb^	0.87 ± 0.03 ^Ac^
		90 d	1.03 ± 0.01 ^Aa^	1.02 ± 0.05 ^Aa^	0.25 ± 0.02 ^Bb^	0.88 ± 0.06 ^Ac^
	*SelT*	30 d	1.00 ± 0.02 ^Aa^	1.01 ± 0.06 ^Aa^	0.44 ± 0.01 ^Ab^	0.78 ± 0.02 ^Ac^
		60 d	1.06 ± 0.03 ^Aa^	1.03 ± 0.01 ^Aa^	0.41 ± 0.00 ^ABb^	0.77 ± 0.02 ^Ac^
		90 d	1.03 ± 0.05 ^Aa^	1.03 ± 0.02 ^Aa^	0.35 ± 0.04 ^Bb^	0.77 ± 0.01 ^Ac^
	*SelU*	30 d	1.02 ± 0.04 ^Aa^	1.04 ± 0.02 ^Aa^	0.75 ± 0.03 ^Ab^	0.92 ± 0.04 ^Ac^
		60 d	1.01 ± 0.03 ^Aa^	1.04 ± 0.03 ^Aa^	0.60 ± 0.02 ^Bb^	0.94 ± 0.02 ^Ac^
		90 d	1.01 ± 0.01 ^Aa^	1.01 ± 0.03 ^Aa^	0.54 ± 0.03 ^Cb^	0.89 ± 0.01 ^Ac^
	*SelPb*	30 d	1.00 ± 0.02 ^Aa^	1.02 ± 0.02 ^Aa^	0.48 ± 0.01 ^Ab^	0.85 ± 0.02 ^Ac^
		60 d	1.01 ± 0.01 ^Aa^	1.04 ± 0.02 ^Aa^	0.38 ± 0.02 ^Bb^	0.84 ± 0.01 ^Ac^
		90 d	1.00 ± 0.03 ^Aa^	1.01 ± 0.03 ^Aa^	0.25 ± 0.03 ^Cb^	0.88 ± 0.02 ^Ac^
	*Sepn1*	30 d	1.02 ± 0.04 ^Aa^	1.04 ± 0.05 ^Aa^	0.34 ± 0.03 ^Ab^	0.83 ± 0.04 ^Ac^
		60 d	1.00 ± 0.02 ^Aa^	1.02 ± 0.03 ^Aa^	0.32 ± 0.02 ^Ab^	0.84 ± 0.03 ^Ac^
		90 d	1.01 ± 0.03 ^Aa^	1.04 ± 0.01 ^Aa^	0.22 ± 0.01 ^Bb^	0.80 ± 0.02 ^Ac^
	*SelP1*	30 d	0.97 ± 0.06 ^Aa^	1.03 ± 0.01 ^Aa^	0.40 ± 0.04 ^Ab^	0.88 ± 0.03 ^Ac^
		60 d	1.02 ± 0.03 ^Aa^	1.05 ± 0.04 ^Aa^	0.26 ± 0.03 ^Bb^	0.87 ± 0.02 ^Ac^
		90 d	1.03 ± 0.02 ^Aa^	1.04 ± 0.03 ^Aa^	0.26 ± 0.03 ^Bb^	0.89 ± 0.04 ^Ac^
	*Sepx1*	30 d	1.04 ± 0.05 ^Aa^	1.01 ± 0.02 ^Aa^	0.48 ± 0.01 ^Ab^	0.71 ± 0.04 ^Ac^
		60 d	0.99 ± 0.04 ^Aa^	1.05 ± 0.02 ^Aa^	0.52 ± 0.14 ^Ab^	0.92 ± 0.03 ^Ac^
		90 d	1.02 ± 0.06 ^Aa^	1.01 ± 0.08 ^Aa^	0.43 ± 0.05 ^Ab^	0.85 ± 0.03 ^Ac^
	*Sepw1*	30 d	0.98 ± 0.04 ^Aa^	1.03 ± 0.02 ^Aa^	0.47 ± 0.02 ^Ab^	0.81 ± 0.02 ^Ac^
		60 d	1.03 ± 0.05 ^Aa^	1.06 ± 0.04 ^Aa^	0.36 ± 0.05 ^Bb^	0.87 ± 0.05 ^Ac^
		90 d	1.05 ± 0.01 ^Aa^	1.03 ± 0.02 ^Aa^	0.37 ± 0.01 ^Bb^	0.84 ± 0.03 ^Ac^
	*Sep15*	30 d	0.99 ± 0.02 ^Aa^	1.03 ± 0.03 ^Aa^	0.47 ± 0.03 ^Ab^	0.77 ± 0.04 ^Ac^
		60 d	1.00 ± 0.01 ^Aa^	1.03 ± 0.01 ^Aa^	0.37 ± 0.06 ^Ab^	0.77 ± 0.01 ^Ac^
		90 d	1.03 ± 0.02 ^Aa^	1.00 ± 0.01 ^Aa^	0.23 ± 0.03 ^Ab^	0.73 ± 0.01 ^Ac^
	*SPS2*	30 d	0.97 ± 0.07 ^Aa^	0.99 ± 0.04 ^Aa^	0.38 ± 0.02 ^Ab^	0.86 ± 0.05 ^Ac^
		60 d	1.00 ± 0.06 ^Aa^	1.04 ± 0.02 ^Aa^	0.36 ± 0.03 ^Ab^	0.83 ± 0.03 ^Ac^
		90 d	0.96 ± 0.04 ^Aa^	0.99 ± 0.05 ^Aa^	0.26 ± 0.09 ^Ab^	0.78 ± 0.07 ^Ac^

## Data Availability

The data presented in this study are available in this article.

## Supplementary Materials

The following supporting information can be downloaded at: https://www.mdpi.com/article/10.3390/antiox13030370/s1. Table S1. The composition of chicken standard commercial diet in our experiment.
