# Supplementary material for: Se Alleviated Pb-Caused Neurotoxicity in Chickens: SPS2-GPx1-GSH-IL-2/IL-17-NO Pathway, Selenoprotein Suppression, Oxidative Stress, and Inflammatory Injury
Source: Antioxidants (Basel). 2024 Mar 18;13(3):370. doi: 10.3390/antiox13030370 (PMC10968013; doi:10.3390/antiox13030370)
Supplement: Supplementary file 1 [file antioxidants-13-00370-s001.zip › antioxidants-2878126-supplementary.pdf]

Table S1 The composition of chicken standard commercial diet in our experiment.

	1-28 days	29-97 days
crude protein (%)	20.5	19.3
moisture (%)	12	13.4
crude fiber (%)	4.3	5.5
crude ash (%)	6.8	7.8
calcium (%)	0.89	0.83
total phosphorous (%)	0.64	0.62
sodium chloride (%)	0.35	0.38
copper (mg/kg)	9.45	9.45
iron (mg/kg)	75.5	75.5
zinc (mg/kg)	45.15	45.15
manganese (mg/kg)	115.41	115.41
iodine (mg/kg)	0.46	0.46
selenium (mg/kg)	0.49	0.49
vitamin A (IU)	2750	2750
vitamin D3 (IU)	410	410
vitamin E (mg/kg)	12	12
vitamin K (mg/kg)	0.6	0.6
vitamin B1 (mg/kg)	1.7	1.7
vitamin B2 (mg/kg)	7.8	4
vitamin B12 (mg/kg)	0.01	0.01
nicotinic acid (mg/kg)	31	31
folic acid (mg/kg)	0.62	0.62
pantothenic acid (mg/kg)	11.5	11.5
biotin (mg/kg)	0.17	0.17
